# The PhyloFacts FAT-CAT web server: ortholog identification and function prediction using fast approximate tree classification

**DOI:** 10.1093/nar/gkt399

**Published:** 2013-05-18

**Authors:** Cyrus Afrasiabi, Bushra Samad, David Dineen, Christopher Meacham, Kimmen Sjölander

**Affiliations:** ^1^QB3 Institute, University of California, Berkeley, Berkeley, CA 94720-1762, USA and ^2^Department of Bioengineering, University of California, Berkeley, Berkeley, CA 94720-1762, USA

## Abstract

The PhyloFacts ‘Fast Approximate Tree Classification’ (FAT-CAT) web server provides a novel approach to ortholog identification using subtree hidden Markov model-based placement of protein sequences to phylogenomic orthology groups in the PhyloFacts database. Results on a data set of microbial, plant and animal proteins demonstrate FAT-CAT’s high precision at separating orthologs and paralogs and robustness to promiscuous domains. We also present results documenting the precision of ortholog identification based on subtree hidden Markov model scoring. The FAT-CAT phylogenetic placement is used to derive a functional annotation for the query, including confidence scores and drill-down capabilities. PhyloFacts’ broad taxonomic and functional coverage, with >7.3 M proteins from across the Tree of Life, enables FAT-CAT to predict orthologs and assign function for most sequence inputs. Four pipeline parameter presets are provided to handle different sequence types, including partial sequences and proteins containing promiscuous domains; users can also modify individual parameters. PhyloFacts trees matching the query can be viewed interactively online using the PhyloScope Javascript tree viewer and are hyperlinked to various external databases. The FAT-CAT web server is available at http://phylogenomics.berkeley.edu/phylofacts/fatcat/.

## INTRODUCTION

FAT-CAT (Fast Approximate Tree Classification) is a web server for protein functional annotation and identification of orthologs. Orthology relationships are used in many bioinformatics analyses, including functional annotation of genomes, phylogenetic profile construction, prediction of protein–protein interaction and phylogenetic studies. The FAT-CAT web server achieves broad taxonomic and functional coverage by making use of pre-calculated phylogenetic trees in the PhyloFacts database (1). FAT-CAT precision is due to the use of hidden Markov models (HMMs) at every node of every tree, allowing highly flexible prediction of function at all levels of a functional hierarchy. PhyloFacts includes trees for many Pfam-A domains (2) and multi-domain architectures (MDAs), with >7.3 M proteins from across the Tree of Life clustered into 92.8 K families. PhyloFacts integrates experimental and annotation data from different resources including SwissProt, the Gene Ontology, Pfam, BioCyc, Enzyme Commission and third-party orthology databases. These data are used to derive a profile of functional descriptions at each subtree node in the PhyloFacts database and to provide functional annotations for user-supplied query sequences.

The input to the FAT-CAT web server is a protein sequence; the maximum sequence length allowed is 2000 amino acids. The FAT-CAT pipeline proceeds through a series of analyses to select a set of subtrees from which candidate orthologs are identified and functional annotations are derived. Four preset pipeline parameters options—high recall, high precision, remote homolog detection and partial sequence search—are designed to handle different types of inputs and to accommodate user preferences for either high recall or high precision. FAT-CAT default parameters are set for high recall, as these are effective on most inputs and are robust to small gene model errors and/or structural variants. High-precision parameter settings restrict predicted orthologs to those that align globally to the query with high sequence identity; we recommend these settings when the query contains a promiscuous domain or has close paralogs. The partial sequence settings are designed for cases where the input is known to be incomplete or represents a splice variant missing one or more exons. Remote homolog detection parameter settings dramatically relax alignment overlap, and percentage identity constraints and are recommended primarily when other parameter settings fail to identify orthologs. Users can also modify individual parameters as desired. Guidelines to selecting from these four preset parameter options and tuning parameters are provided in the Online Help and in the Supplementary Materials.

Program outputs are organized into a web page with separate tabbed views for family matches, predicted orthologs, functional annotations derived from orthologs, phylogenetic trees and other data. PhyloFacts trees matching the query can be viewed interactively online using the PhyloScope Javascript tree viewer and are hyperlinked to various external databases providing functional annotations for family members. Users can also explore the more remote evolutionary relationships on the Distant Clades tab to obtain additional clues to function and/or structure. The Job Summary tab stores all program inputs including the query sequence and all parameter settings. Downloads are provided for key data.

## MATERIALS AND METHODS

### The PhyloFacts database

PhyloFacts 3.0 includes >7.3 M proteins from 99 K unique taxa across Bacteria, Archaea and Eukarya clustered into 92.8 K protein families. The number of sequences per genome in PhyloFacts follows a power law: 95.6 K taxa have ≤100 sequences, 1.2 K have between 101 and 1 K, and 2.2 K genomes have >1 K sequences each. PhyloFacts families represent both individual Pfam domains and MDAs (multi-domain architectures, homology clusters where sequences align globally); sequences are drawn from the UniProt database, including both whole and partly sequenced genomes. Each family has a multiple sequence alignment (MSA), phylogenetic tree, predicted orthologs, HMMs and annotation data drawn from various sources. The PhyloFacts 3.0 library construction pipeline differs slightly from the pipeline used in release 2.0 (1): first, PhyloFacts 3.0 includes trees for Pfam domains (described later in the text); second, PhyloFacts 3.0 trees are constructed using FastTree (3). PhyloFacts family pages and the overall website have been redesigned for easier navigation and interpretation.

#### PhyloFacts MDA families

We use the FlowerPower algorithm (4) to cluster sequences sharing a common MDA. FlowerPower is an iterated homology clustering algorithm that uses Subfamily Classification in Phylogenomics (SCI-PHY) (5) to identify subfamilies and subfamily HMMs to select and align new sequences. In each iteration, as new sequences are retrieved and aligned, FlowerPower examines the alignment of candidate family members for agreement with the family consensus structure. The resulting cluster of homologs has both high precision and recall in clustering sequences into MDA classes (4).

#### PhyloFacts-Pfam

We provide trees for Pfam domains, and orthologs derived based on analysis of these trees, for two reasons. First, Pfam domains provide important clues to the functions of proteins. Second, domain-based phylogenies are often better resolved than those based on multiple sequence alignments requiring sequences to align globally. A stringent requirement of global alignment can reject actual orthologs having relatively small gene model errors. Constructing trees for Pfam domains (requiring only local matches within proteins) allows us to relax these constraints, increasing taxon sampling density and improving the resolution of the phylogenetic tree topology. Although individual sequences in a homology cluster may have differences in MDA, sequences that share the same MDA tend to cluster into subtrees, even when the alignment used as the basis for the phylogenetic reconstruction is restricted to the single domain they all share in common. The result is an improvement in ortholog identification recall and precision (6).

#### Orthology prediction methods included in FAT-CAT

We use two methods developed by our laboratory [Kerf and PHOG (7)] and retrieve orthology data from third-party orthology databases [OMA (8) and OrthoMCL (9)]. PHOG is a phylogenomic orthology prediction method that uses phylogenetic tree distances to identify duplication events on PhyloFacts trees from which orthologs can be identified; a tree distance threshold controls precision and recall. A tree-distance threshold of zero [PHOG-T(0)] corresponds to a highly conservative identification of super-orthologs (10), i.e. all sequences in the subtree are each other’s orthologs, and no duplication events are allowed subsequent to a speciation event. Comparisons on a benchmark data set of manually curated orthologs showed PHOG to be competitive with OrthoMCL DB (9) and InParanoid (11) for precision and recall (7). The Kerf algorithm uses a simple sequence identity threshold to cut a tree into subtrees based on a tolerated sequence divergence; by default, we use a cutoff of 70% sequence identity. (In other words, using a Kerf cutoff of 70% identity produces a cut of a tree into subtrees such that no pair of sequences in any subtree has <70% sequence identity.)

Third-party orthology data are overlaid on PhyloFacts trees, and a subtree-bracketing protocol is used to find maximal subtrees meeting the following criteria: sequences in the subtree are either unlabeled (by that orthology database) or belong to a single orthology group, and at least one member of the left and right child nodes descending from the subtree root have been assigned to that orthology group by the orthology database. This subtree bracketing protocol produces a set of sequences that are consistent with the third-party database but which may include sequences that are not explicitly labeled by that database. We currently include data from OMA (8) and OrthoMCL (9) and use these data for subtree bracketing in FAT-CAT. Supplementary Figure S2 in the Supplementary Materials illustrates subtree bracketing.

### The FAT-CAT web server pipeline

**Figure 1.**
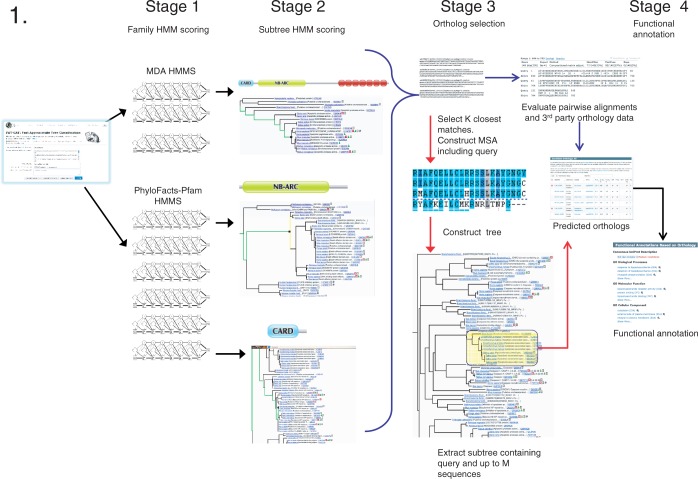
The FAT-CAT pipeline. The FAT-CAT pipeline starts with the submission of a protein sequence and parameter selection and proceeds through family and subtree HMM scoring to ortholog identification and functional annotation. The FAST-CAT variant differs from the default FAT-CAT pipeline in Stage 3 (indicated by red arrows). In Stage 1, the query is scored against family HMMs in the PhyloFacts database for proteins sharing the same multi-domain architecture (MDA) (shown at top) and HMMs constructed for Pfam domains (shown at bottom). Families meeting Stage 1 criteria (*E*-value and alignment statistics) are passed to Stage 2. In this toy example, PhyloFacts trees for two Pfam domains and a tree for the MDA meet Stage 1 criteria and are passed to Stage 2. In Stage 2, we obtain an approximate phylogenetic placement of the query in each tree by scoring all the HMMs in the tree. The subtree node corresponding to the top-scoring HMM is examined to determine its suitability as a source of orthologs to the query: Stage 2 parameters include the query-subtree HMM score and alignment statistics and whether the subtree appears to be restricted to orthologs. For each top-scoring node that meets these criteria, we identify a (typically larger) enclosing clade supported by one or more orthology methods. Enclosing clades are passed to Stage 3 for ortholog identification. In Stage 3, FAT-CAT and FAST-CAT diverge. FAT-CAT (blue arrows) evaluates the pairwise alignment between the query and each sequence and identifies all supporting evidence supporting the orthology. FAST-CAT (red arrows) avoids much of this computational complexity by using a fast k-tuple comparison to select the most similar sequences from the enclosing clade, constructing an multiple sequence alignment (MSA) including the query using MAFFT, estimating a phylogenetic tree using FastTree, and extracting a subtree of the phylogenetically closest sequences (i.e. based on tree distance to the query). Alignment analysis can then be restricted to this smaller subset based on the multiple sequence alignment. Sequences meeting these criteria are then passed to Stage 4. In Stage 4, we derive a weighted consensus functional annotation for the query based on orthologs selected in Stage 3. Annotations from close orthologs are given higher weight than those from more distant orthologs, and manually curated annotations are given higher weight than those that are derived computationally.

We provide two main pipelines: FAT-CAT and FAST-CAT. FAT-CAT analyses are computationally expensive so that results may take one or more hours to complete. To accommodate users preferring faster results, we provide a variant of the pipeline, FAST-CAT, which returns results for most queries in minutes. The FAT-CAT pipeline (including the FAST-CAT bypass) is displayed in Figure 1.

The input to the web server is a protein sequence; inputs up to 2000 amino acids are allowed. Each stage in the pipeline has parameters to control the precision and recall of results and to accommodate different types of sequence inputs; all parameters can be modified by the user. Details on pipeline parameter settings for the four preset options are provided in the Supplementary Materials; guidelines are provided in the Online Help. In Stage 1, we score the query against HMMs located at the root of each family tree; families meeting HMM *E*-value and other criteria are forwarded to Stage 2. In Stage 2, we score the query against all HMMs in each tree; the top-scoring node is identified, and additional criteria are evaluated including subtree HMM *E*-value, query-HMM alignment statistics and characteristics of the subtree (e.g. whether the subtree is supported by internal or third-party orthology methods). Subtrees that meet these criteria are passed to Stage 3. In Stage 3, we identify an ‘enclosing clade’ including (but potentially larger than) the top-scoring subtree node from which a set of candidate orthologs is extracted. Candidate orthologs are aligned to the query, and the alignment is evaluated against sequence identity and overlap criteria. Because the UniProt sequence database (from which PhyloFacts draws sequences for trees) is not restricted to whole genomes, it is not uncommon to have multiple representatives for the same gene in the same species (e.g. representing different isoforms, splice variants or different gene models). These sequences often show up in PhyloFacts trees as apparent clusters of inparalogs, but they represent alternate isoforms or gene models for the same gene. We handle these sequences as follows: first, we cluster sequences from the same genome based on sequence identity to the query and select single representatives of each cluster (choosing a SwissProt sequence if one is available); other cluster members are linked on the results page. In cases where an enclosing clade has included two or more clusters from the same genome and both meet the alignment overlap and percentage identity thresholds specified by the user, the cluster with higher percentage identity will be designated as orthologous, and the group with lower percentage identity will be designated as paralogous. In Stage 4, we transfer annotations from orthologs meeting defined criteria, weighting annotations according to the evolutionary distance between the query and ortholog and the support for the annotation being transferred.

#### The FAST-CAT pipeline

FAST-CAT and FAT-CAT are identical in the first two stages of family and subtree HMM scoring and then again in the final Stage 4, where functional annotations are derived for the query. They differ only in Stage 3, when orthologs are identified: FAT-CAT includes additional quality assurance analyses including integration of third-party orthology data over each phylogenetic tree passing Stage 2.

### Benchmarking FAT-CAT accuracy

We performed two sets of experiments to evaluate the FAT-CAT web server for accuracy. First, we evaluated the subtree-HMM-based assignment of sequences for precision of ortholog identification (i.e. Stage 2 of the FAT-CAT web server pipeline). Second, we compared the FAT-CAT web server pipeline as a whole, comparing the orthologs predicted by FAT-CAT to orthologs predicted by the major orthology web servers. Both sets of experiments are summarized later in the text; details are in the Supplementary Materials, Benchmarking Experiments.

#### Benchmarking the FAT-CAT web server relative to other orthology web servers

We compared the FAT-CAT web server against other major orthology web servers on a small benchmark data set of seven proteins: six eukaryotic and one bacterial. The first test sequence was from rice, a member of the large receptor-like protein superfamily involved in innate immunity and development (12). Five test sequences were vertebrate (one chicken and four human) sequences drawn from the manually curated SwissProt database and represented large multi-gene families with many distinct functional subtypes (G protein-coupled receptors, ion channels, transcription factors, Toll-like receptors and inorganic pyrophosphatases). The last test sequence was drawn from the human oral microbiome. Four of the seven test sequences had promiscuous domains, included to allow us to evaluate the robustness of orthology web servers to these data. We compared predicted orthologs from FAT-CAT (using both the High Recall and High Precision settings) against results from seven other orthology web servers: KEGG (13), PhylomeDB (14), eggNOG (15), OMA (8), OrthoMCL DB (9), InParanoid (11) and OrthoDB (16). FAT-CAT High Precision settings provided the highest accuracy overall, with OMA, PhylomeDB and FAT-CAT High Recall also providing excellent results. All other orthology web servers show a propensity to include paralogs and/or sequences with different MDAs among their proposed orthologs. Details are in the Supplementary Materials, Benchmarking Experiments: Case Studies.

#### Ortholog identification using subtree HMMs

The FAT-CAT pipeline, shown in Figure 1, proceeds through a series of analyses to predict orthologs. In Stage 2 of the pipeline, sequences are scored against HMMs placed at all subtree nodes (including leaf nodes) and classified to the top-scoring subtree. We evaluated the precision of this subtree-HMM classification protocol using leave-1-out experiments. FAT-CAT subtree-HMM classification is related closely to our previous work on subfamily HMM-based functional classification (5) in which we constructed HMMs for a discrete set of subtree nodes [defined using the SCI-PHY subfamily identification algorithm (5,17)]. Although HMM scores across families are notoriously non-comparable, within a family defined by a multiple sequence alignment where all sequences are roughly the same length, HMM scores are actually comparable. In a series of leave-1-out experiments, we showed that assigning a sequence to the top-scoring subfamily HMM had near-perfect precision (error rate ∼1%). The basic principles are the same in FAT-CAT, except that we score the query against HMMs at every node in the tree and classify the sequence to the top-scoring subtree. To confirm the accuracy of the FAT-CAT subtree-HMM classification, we repeated a similar set of experiments, using leave-1-out experiments to classify test sequences to orthology groups defined by a strict consensus of three orthology methods: Kerf (requiring a minimum of 70% identity), OMA and OrthoMCL. Results are nearly identical: on a data set of 83 non-homologous families from across the Tree of Life, FAT-CAT classification of withheld sequences to the top-scoring subtree has >99% precision at phylogenetic placement within orthology groups. Details are in the Supplementary Materials, Benchmarking Experiments: Ortholog Identification using Subtree HMMs.

## RESULTS

Users can either provide an email address and receive a URL subsequently when the pipeline has completed or bookmark the results page. Some results return quickly, particularly if the FAST-CAT option has been selected, whereas others may take hours to complete.

**Figure 2.**
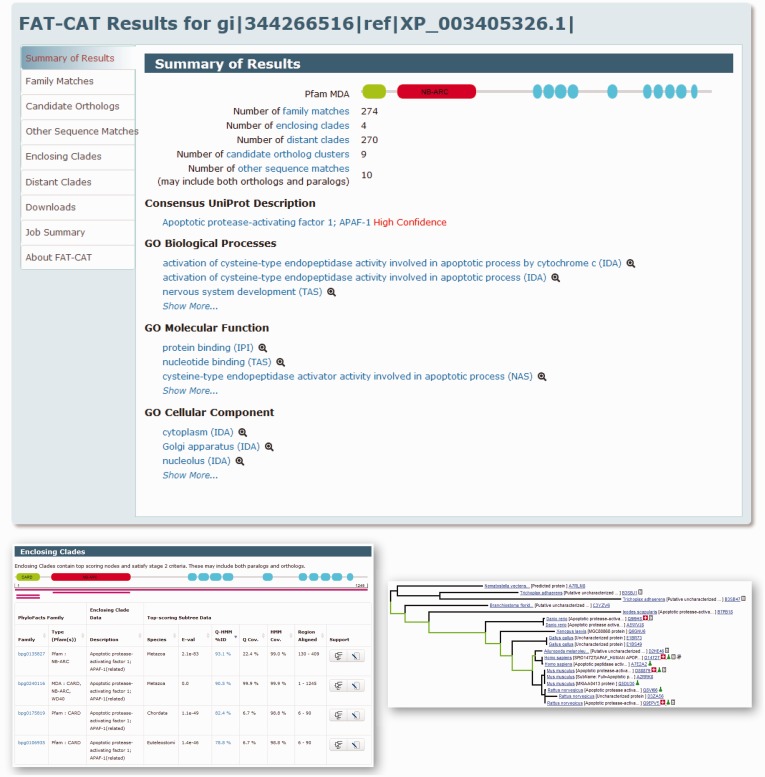
Example FAT-CAT results. The query sequence (gi|344266516|ref|XP_003405326.1) is a predicted apoptotic protease-activating factor 1 from *Loxodonta africana* (African elphant). Top: the Summary of Results page, presenting an overview of results, including the Pfam MDA for the query produced by scanning Pfam-A HMMs. The FAT-CAT pipeline identified 274 families matching Stage 1 criteria and orthologs from nine different genomes (candidate ortholog clusters). Predicted functional annotations for the query derived from orthologs satisfying Stage 3 criteria are displayed. The Job Summary tab displays the input sequence and all pipeline parameters. Bottom left: Enclosing clades passing Stage 2 criteria, displaying matches along the entire MDA as well as to individual Pfam domains. Bottom right: Clicking on the tree icon in the data table in the Enclosing Clade tab displays the tree for an enclosing clade, highlighting the path from the root of the enclosing clade to the top-scoring node. The Phyloscope viewer allows users to view which sequences have experimental support for their annotations and provides links to external databases and to internal PhyloFacts pages. Results can be viewed online at http://phylogenomics.berkeley.edu/phylofacts/fatcat/2616/.

Sample results are shown in Figure 2. Candidate orthologs satisfying Stage 3 criteria are displayed in the ‘Candidate Orthologs’ tab, whereas sequences in one or more enclosing clades that fail one or more criteria are displayed in the ‘Other Sequence Matches’ tab. UniProt consensus functional descriptions, along with confidence scores derived from the annotation source (e.g. weighting SwissProt annotations higher than those from TrEMBL, and giving higher weight to closely related orthologs than to those with lower sequence identity) are shown along with Gene Ontology annotations and evidence codes retrieved from orthologs. Drill-down capabilities are provided to view the provenance of the annotations.

## DISCUSSION

Although the specifics differ, orthology prediction web servers are variations on the same basic theme: orthology relationships are first derived offline for selected genomes and stored in a database; the web server provides a mechanism (most commonly, BLAST) to assign the query to one or more pre-computed orthology groups. We define orthology web servers as those that can classify sequences that are not already in a database. By contrast, an orthology database may have pre-computed orthologs, but if it only provides a look-up functionality (e.g. by sequence accession), it is not an orthology web server.

Orthology web servers can be differentiated on five main criteria: (i) the taxonomic range and density of coverage for sequences included in the database; (ii) the approach used to define orthology groups; (iii) how input sequences are classified to orthology clusters; (iv) alignment statistics and other evidence provided to the user to support a predicted orthology; and (v) whether a functional annotation is derived based on that orthology.

Restrictions on taxonomic range and density of coverage limit the sequences a web server can effectively classify as well as the orthologs it can identify. Orthology prediction web servers with broad taxonomic coverage include FAT-CAT (7.3 M sequences across 99 K unique taxa, of which 2.2 K have >1 K sequences each), KEGG (10 M sequences across 2.4 K species) (13), PhylomeDB (8.6 M sequences across 1.5 K species) (14), eggNOG (4.4 M sequences across 1.1 K species) (15), OMA (6.2 M sequences across 1.3 K species) (8), OrthoMCL DB (1.4 M sequences across 150 species) (9) and OrthoDB (1.4 M sequences across 1.3 K species) (16).

Phylogenomic methods for defining orthology relationships (i.e. methods that explicitly reconstruct the evolutionary history of a multi-gene family to differentiate between orthologs and paralogs) have been observed to have higher precision than graph-based methods based on pairwise genome comparisons between whole genomes (18). Of the orthology web servers discussed here, PhylomeDB and PhyloFacts use phylogenetic tree analysis to define orthology groups, whereas the other resources use graph-based approaches.

Most orthology web servers use BLAST to match a user query to pre-defined orthology groups and provide an ordered list of candidate homologs, which the user can then investigate to find orthologs to those matches. FAT-CAT is unique in using subtree HMMs to place sequences at different levels of functional and evolutionary hierarchies. When sequences included in a phylogeny are closely related to the query, the top-scoring HMM will be close to the leaves, within a pre-defined orthology group. However, when a sequence is more distantly related, the top-scoring HMM will often be higher in the tree, above the level of pre-defined orthology groups. This phylogenetic placement provides a greater perspective on the reliability of an inferred evolutionary relationship, avoiding errors associated with overly precise classifications. FAT-CAT also uses the user-defined alignment overlap and percentage identity criteria to separate candidate orthologs from other sequences. These alignment analyses are the primary reason why FAT-CAT is successful at handling sequences containing promiscuous domains so that predicted orthologs are restricted to those that share the same MDA.

The level of information provided to the user about the query-ortholog match is another important feature of an orthology web server. Detailed alignment statistics, particularly the sequence identity and fractional overlap (of both the query and the candidate ortholog), are critical, as, in general, there is always a closest match in a database, but the closest match may not be orthologous. For instance, if the alignment between the query and a proposed ortholog is restricted to a local region, orthology is questionable even if the *E*-value is significant. Most (but not all) of the web servers analyzed here provide *E*-values between the query and candidate orthologs; only KEGG, PhylomeDB, OrthoMCL and FAT-CAT provide detailed alignment statistics including percentage identity and overlap data.

Finally, FAT-CAT derives a functional annotation for the query sequence along with drill-down capability to view details on the provenance supporting the functional annotation.

The small data set presented here includes proteins that challenge even the best methods, combining lineage-specific expansions and losses and domain architecture rearrangements. Given the central role played by orthology prediction in both genome annotation and phylogenetic studies, there is a great need for the scientific community as a whole to contribute to the development of appropriate orthology benchmarking data sets, such as those under development by the Quest for Orthologs consortium (19).

As our results show, FAT-CAT provides a combination of high precision in discriminating between paralogs and orthologs and robustness to promiscuous domains. However, the behind-the-scenes computations required to provide this precision comes at a cost: FAT-CAT is much slower than other orthology web servers. We are working to reduce the computational complexity of our pipeline; in the meantime, we recommend the use of our FAST-CAT version, available on the same input form. FAST-CAT has most of the functionality of FAT-CAT but returns results in a fraction of the time.

Finally, we propose that controversies over the utility of orthologs for functional inference may be due, at least in part, to the types of coarse-grained clustering of homologs (some of which have only partial matches) produced by some orthology methods. If ‘orthologs’ have only partial homology or are actually distantly related, why should we expect them to have the same function? Phylogenomic methods of orthology prediction, such as provided by FAT-CAT and PhylomeDB, have high precision in differentiating between orthologs that share the same function and homologs whose functions have diverged. The combination of protein structural analyses, including Pfam domain-based phylogenies, provides an additional axis for improving the resolution of orthology prediction (6). Despite the challenges, ortholog identification remains a powerful tool for functional inference and is essential in evolutionary studies.

## SUPPLEMENTARY DATA

Supplementary Data are available at NAR Online: Supplementary Table 1, Supplementary Figures 1–2 and Supplementary Documents 1–2.

## FUNDING

Department of Energy, Biological and Environmental Research. Funding for open access charge: Department of Energy [DE-SC0004916 to K.S.].

*Conflict of interest statement*. None declared.
